# TILT: Time-Lapse Imaging Trial—a pragmatic, multi-centre, three-arm randomised controlled trial to assess the clinical effectiveness and safety of time-lapse imaging in in vitro fertilisation treatment

**DOI:** 10.1186/s13063-020-04537-2

**Published:** 2020-07-01

**Authors:** Priya Bhide, Arasaratnam Srikantharajah, Doris Lanz, Julie Dodds, Bonnie Collins, Javier Zamora, David Chan, Shakila Thangaratinam, Khalid S. Khan

**Affiliations:** 1 Barts Research Centre for Women’s Health, Institute of Population Health Sciences, Queen Mary University of London, London, UK; 2 Homerton Fertility Centre, Homerton University Hospital, London, UK; 3 Centre for Reproductive Medicine, St Bartholomew’s Hospital, London, UK; 4 Clinical Biostatistics Unit, Hospital Ramón y Cajal (IRYCIS), CIBER Epidemiology and Public Health, Madrid, Spain; 5 Assisted Reproductive Technology Unit, Department of Obstetrics and Gynaecology, Faculty of Medicine, The Chinese University of Hong Kong, Sha Tin, Hong Kong SAR; 6 Institute of Metabolism and Systems Research, WHO Collaborating Centre for Women’s Health, University of Birmingham, Birmingham, UK; 7 Department of Preventive Medicine and Public Health, University of Granada, Granada, Spain

**Keywords:** Time-lapse imaging, In vitro fertilisation, Live birth, Fertility, Assisted conception

## Abstract

**Background:**

Subfertility is a common problem for which in vitro fertilisation (IVF) treatment is commonly recommended. Success rates following IVF are suboptimal and have remained static over the last few years. This imposes a considerable financial burden on overstretched healthcare resources. Time-lapse imaging (TLI) of developing embryos in IVF treatment is hypothesised to improve the success rates of treatment. This may be either by providing undisturbed culture conditions or by improving the predictive accuracy for optimal embryo selection from a cohort of available embryos. However, the current best evidence for its effectiveness is inconclusive.

**Methods:**

The time-lapse imaging trial is a pragmatic, multi-centre, three-arm parallel-group randomised controlled trial using re-randomisation. The primary objective of the trial is to determine if the use of TLI or undisturbed culture in IVF treatment results in a higher live birth rate when compared to current standard methods of embryo incubation and assessment. Secondary outcomes include measures of clinical efficacy and safety. The trial will randomise 1575 participants to detect an increase in live birth from 26.5 to 35.25%.

**Discussion:**

In the absence of high-quality evidence, there is no current national guidance, recommendation or policy for the use of TLI. The use of TLI is not consistently incorporated into standard IVF care. A large, pragmatic, multi-centre, trial will provide much needed definitive evidence regarding the effectiveness of TLI. If proven to be effective, its incorporation into standard care would translate into significant clinical and economic benefits. If not, it would allow allocation of resources to more effective interventions.

**Trial registration:**

ISRCTN registry ISRCTN17792989. Prospectively registered on 18 April 2018

## Administrative information

Note: the numbers in curly brackets in this protocol refer to SPIRIT checklist item numbers. The order of the items has been modified to group similar items (see http://www.equator-network.org/reporting-guidelines/spirit-2013-statement-defining-standard-protocol-items-for-clinical-trials/).

**Table:** 

Title {1}	TILT: Time-lapse Imaging Trial: A pragmatic, multi-centre, three-arm randomised controlled trial to assess the clinical effectiveness and safety of time-lapse imaging in in-vitro fertilisation treatment.
Trial registration {2a and 2b}.	ISRCTN registry, ISRCTN17792989 Date of registration 18/04/2018 http://www.isrctn.com/ISRCTN17792989
Protocol version {3}	TILT protocol v7.0, 04/Sep/2019
Funding {4}	Barts Charity, UK, Funder Reference Number MGU0374 Pharmasure Ltd. (UK)
Author details {5a}	Priya Bhide^1,2^, Arasaratnam Srikantharajah^2^, Doris Lanz^1^, Julie Dodds^1^, Bonnie Collins^3^, Javier Zamora^1,4^, David Chan^5^ Shakila Thangaratinam^6^, Khalid S. Khan^7^ ^1^ Barts Research Centre for Women’s Health, Institute of Population Health Sciences, Queen Mary University of London. ^2^ Homerton Fertility Centre, Homerton University Hospital, London, UK. ^3^ Centre for Reproductive Medicine, St Bartholomew’s Hospital, London, UK. ^4^ Clinical Biostatistics Unit. Hospital Ramón y Cajal (IRYCIS). CIBER Epidemiology and Public Health, Madrid. ^5^ Assisted Reproductive Technology Unit, Department of Obstetrics and Gynaecology, Faculty of Medicine, The Chinese University of Hong Kong, Hong Kong SAR. ^6^ Institute of Metabolism and Systems Research, WHO Collaborating Centre for Women’s Health, University of Birmingham, Birmingham, UK. ^7^ Department of Preventive Medicine and Public Health, University of Granada, Spain.
Name and contact information for the trial sponsor {5b}	UK Sponsor: Queen Mary University of London UK Sponsor contact: Dr. Mays Jawad, Research Governance Operations Manager, Joint Research Management Office, 5 Walden Street, London, E1 2EF Phone: 020 7882 7275 Email: research.governance@qmul.ac.uk Co-sponsor: The Chinese University of Hong Kong Office of Research and Knowledge Transfer Services Room 301, Pi Ch’iu Building Shatin, New Territories Hong Kong SAR
Role of sponsor {5c}	The sponsor and the funder have no role in the study conduct, analysis and interpretation of the findings, and dissemination of the results.

## Introduction

### Background and rationale {6a}

#### Background

Subfertility is a common problem affecting 1 in 7 couples. In vitro fertilisation/intracytoplasmic sperm injection (IVF/ICSI) is a fertility treatment often recommended to these couples. It involves fertilisation of the oocytes with sperm in the laboratory to create embryos. The best one or two embryos are then selected for transfer back into the womb.

##### Increasing demand for treatment

The number of women having IVF has seen a steady increase over the years from 14,057 in 1992 to 52,288 in 2014 in the UK, with the latest national report showing approximately 68,000 treatment cycles performed annually in the UK in 2014 [1]. The National Institute of Health and Clinical Excellence (NICE) recommends 3 NHS-funded treatment cycles per couple [2].

##### Static success rates

Success rates following IVF are suboptimal and have remained static over the last few years. European registers report an average of 32% clinical pregnancy rate in fresh (excluding treatment with frozen embryos) IVF cycles (2013), and in the UK, the Human Fertilisation and Embryology Authority (HFEA) reports a 26.5% live birth rate for fresh IVF cycles for 2013 [1]. European registries and UK data show no improvements in live birth rate over the last 3 years.

##### Economic considerations

Forty per cent of IVF treatments in the UK are funded by the NHS at the average tariff of £3500 per treatment cycle, imposing a considerable financial burden on overstretched NHS resources.

##### Multiple births

Transferring two embryos back into the womb (double embryo transfer [DET]) is a common practice in IVF/ICSI in order to maximise the chance of pregnancy. DET however results in a much higher percentage of multiple pregnancies, between 24 and 36%, as compared to single embryo transfers [1]. Multiple pregnancies are associated with an increase in perinatal morbidity and mortality and increased maternal morbidity. The short- and long-term health burden from this increased morbidity has implications for the children and parents, as well as the NHS and other publicly funded services. Transferring only a single embryo back into the womb (elective single embryo transfer [e-SET]) can reduce the risk of multiple pregnancies but has the potential to reduce success rates. However, e-SET is used in only around 30% of all transfers, and as a result, multiple pregnancy rates following IVF/ICSI are still approximately 15% compared to 1.6% in natural conceptions [1].

Pregnancy rates following IVF/ICSI are positively correlated with appropriate and optimal embryo selection from the available cohort of embryos. The current methods of embryo selection have poor predictive accuracy. If the prediction of the implantation potential of embryos could be improved, this would not only improve the overall live birth rates following treatment but also facilitate embryo selection for single rather than double embryo transfer to reduce multiple birth rates. Embryo selection to improve pregnancy rates remains a significant challenge in IVF. NICE [2] and a recent commentary from the British Fertility Society (BFS) and Association of Clinical Embryologists (ACE) [3] recommend further research to improve embryo selection to facilitate single embryo transfers.

#### Rationale

Time-lapse imaging (TLI) of the developing embryos is a new technology available in IVF/ICSI laboratories. It involves digital imaging of the developing embryos at intervals of every 5–15 min from the time of IVF/ICSI up to day 5 of culture. These images are used to create a time-lapse sequence of embryo development visualised on an external monitor using specialised computer software. TLI systems may confer a dual advantage over conventional systems. They allow morphological assessment of embryos without the need for removal of embryos from the incubators, giving a potential advantage of undisturbed culture conditions for the embryos. TLI also allows the embryologists to gain more information about embryo development, especially transient events which may be missed by traditional assessment methods by the generation of variables called ‘morphokinetic parameters’. By providing undisturbed culture conditions and/or the addition of morphokinetic parameters to standard methods of embryo selection, TLI is hypothesised to improve the predictive accuracy for optimal embryo selection from a cohort of available embryos and hence the success rates for treatment.

#### Existing research

A systematic review on this topic was published in February 2015 by the Cochrane Collaboration [4]. This review aimed to assess the effect of TLI compared with standard practice on live birth, clinical pregnancy, miscarriage and stillbirth rates. The authors acknowledged the effect of two variables in the TLI systems, undisturbed culture and morphokinetic parameters, and aimed to assess not only the overall impact of the system but the relative and independent contributions of these two variables. This review included only three randomised controlled trials (RCTs), with a total of 994 participants. Only one trial assessed live birth rate, and due to the small sample size (*n* = 76), results were imprecise and inconclusive (OR [TLI vs. standard practice] 1.1, 95% CI 0.5 to 2.7, moderate-quality evidence). Likewise, results regarding clinical pregnancy, miscarriage and stillbirth rates were also inconclusive; this was partly because the two moderate quality studies had very small sample sizes (*n* = 76, *n* = 62), while the third, larger study (*n* = 856) was assessed as having a high risk of bias. Two of the above three studies assessed the impact of TLI due to undisturbed culture only, without the addition of morphokinetic parameters to standard care. This showed no conclusive differences in live birth, miscarriage and stillbirth rates. The third, larger study compared the combined effect of morphokinetic parameters and undisturbed culture to standard care, which showed no conclusive differences in clinical pregnancy rates. An update to this review was published in 2018 and presented the same conclusions [5].

A recent meta-analysis by Chen et al. [6] reiterated the findings of the Cochrane review. A study by Goodman et al. [7] showed no significant advantage of the addition of morphokinetic parameters to undisturbed culture, when all embryos were cultured in a closed system. Again, this trial reported clinical pregnancy rate rather than live birth rate as its primary outcome with no health economic evaluation.

In conclusion, the current best evidence has provided inconclusive evidence regarding the effectiveness of time-lapse imaging. In the absence of high-quality evidence, there is no current national guidance, recommendation or policy for the use of time-lapse imaging in IVF/ICSI. The use of time-lapse imaging is not consistently incorporated into the standard plan of IVF/ICSI in IVF centres across the UK. It is however available at many centres, where it is offered to couples usually at an extra charge. A large, pragmatic, multi-centre, trial would provide much needed definitive evidence regarding the effectiveness of TLI for patients.

#### Potential risks and benefits

There is no added risk foreseen by using time-lapse imaging technology. Any devices used in this trial are CE-marked and used within their certified indication. Time-lapse imaging requires exposing embryos to infrared light for about 15 ms every 5 to 15 min; this light exposure has been tested and found to be equivalent to light exposure using standard incubators [8]. The hypothesised benefit of TLI is that it will improve the rate of IVF/ICSI success, either by improving selection through analysis of morphokinetic parameters or by allowing embryos to develop in an undisturbed culture.

#### Why this research is urgently needed now

##### Inconclusive and inadequate evidence

As stated above, the current best evidence for the use of time-lapse imaging in IVF/ICSI is inconclusive. Previous RCTs have typically used the clinical pregnancy rate as a surrogate outcome for the live birth rate, which remains the most relevant outcome for patients, clinicians and policymakers. Furthermore, the long-term safety of this procedure is unknown.

##### Clinical equipoise

In preparation for the trial, a survey was sent out to lead clinicians and embryologists across the UK to assess their views on the use, effectiveness and the need for conclusive evidence on the use of TLI. Of those who responded, 100% agree that a well-planned randomised controlled trial is essential. A state of clinical equipoise was registered by 70% of the respondents. We also surveyed patients’ opinion through our collaborators Fertility Network UK. Seventy-three per cent of 80 respondents were in a state of participant equipoise, and 100% agree that a definitive conclusive trial is necessary.

##### Widespread use

TLI in IVF/ICSI is commercially available, extensively marketed and widely introduced in IVF clinics in spite of clinical equipoise and inconclusive evidence regarding its effectiveness. The systems impose a considerable additional expense to clinics in the form of capital costs, running costs, consumables and embryology time. Couples who pay for the treatments are charged between £350 and 1000 for TLI, which is almost an additional 25% of the cost of the standard IVF/ICSI treatment. In the absence of high-quality evidence and rapidly slipping clinical equipoise, it is necessary to urgently perform a large randomised controlled trial to confirm or refute the benefit of time-lapse imaging in IVF/ICSI.

### Objectives {7}

#### Primary objectives

The primary objective of the trial is to determine if the use of TLI or undisturbed culture in IVF/ICSI treatment results in a higher live birth rate when compared to current standard methods of embryo incubation and assessment.

#### Secondary objectives

The trial aims to answer the following questions, and the secondary objectives of the trial are as follows:

1. To obtain measures of the clinical effectiveness of TLI/undisturbed culture:
  Are clinical pregnancy rates and implantation rates increased with the use of TLI/undisturbed culture in IVF/ICSI treatment?
  Does the use of TLI/undisturbed culture increase the number of women having an elective single embryo transfer?
2. To obtain measures of clinical safety of TLI/undisturbed culture:
  Does the use of TLI/undisturbed culture decrease the incidence of multiple births following IVF/ICSI treatment?
  Does the use of TLI/undisturbed culture decrease the incidence of miscarriages following IVF/ICSI treatment?
  Are stillbirths and major congenital abnormalities at birth decreased with the use of TLI/undisturbed culture in IVF/ICSI treatment?

### Trial design {8}

#### Study design

The trial is a pragmatic, multi-centre, three-arm parallel-group randomised controlled trial using re-randomisation.

The hypothesised benefit of the TLI systems may be due to either a closed undisturbed culture system or the use of morphokinetic parameters for embryo selection or both. Hence, a three-arm study design is necessary to answer the research question and define the contributions of both the variables involved.

The trial will compare the results of the following:

1. Intervention 1: incubation and assessment of embryos using TLI systems (morphokinetic parameters + undisturbed culture + morphological assessment)
2. Intervention 2: incubation of embryos in undisturbed culture and standard embryo assessment (undisturbed culture + morphological assessment)
3. Control: standard care (morphological assessment alone)

The design of this trial will be pragmatic. It will allow centres to use a TLI system of their choice. The trial tests the basic premise that undisturbed culture and/or addition of morphokinetic parameters to standard embryo assessment improves outcomes in IVF/ICSI treatment. It does not aim to test nor validate individual TLI systems and algorithms. The trial will use the manufacturer’s algorithm for morphokinetic parameters as a minimum standard to ensure quality control and external validity to the study. It will allow centres to develop more sophisticated centre-specific algorithms by incorporating further parameters into the basic model recommended by the manufacturer. It is accepted that clinical protocols for IVF and laboratory conditions will vary with each centre. All clinical and laboratory elements of IVF/ICSI treatment apart from the randomised intervention will be carried out according to local centre protocols, ensuring only that there is no variation across the three study arms in culture medium and incubator settings (temperature, CO_2_ and O_2_ levels) within each centre.

Couples having IVF/ICSI treatment will be eligible for the trial. Eligible participants will be screened in the fertility clinics and invited to participate in the trial. Following informed consent from both partners, participants will be randomised in a 1:1:1 ratio to one of the three arms: TLI, undisturbed culture or standard care. Women will be followed up until either a negative pregnancy test 2 weeks after embryo transfer or to the end of their pregnancy following a positive pregnancy test.

A consort flow chart is attached as Additional file 1.

## Methods: participants, interventions and outcomes

### Study setting {9}

This is a multi-centre trial being carried out at centres across the UK and internationally. Participants will be recruited at UK HFEA-licenced as well as international fertility centres that have full IVF/ICSI laboratory services available.

### Eligibility criteria {10}

#### Inclusion criteria

The inclusion criteria are broad in keeping with the latest NICE guidelines (2013) for NHS-funded IVF/ICSI treatment.

Participants undergoing IVF/ICSI treatment and:

1. The woman is between 18 and 42 years of age at the time of consent
2. The male partner is at least 18 years of age at the time of consent
3. Receiving the first, second or third IVF/ICSI treatment cycle
4. Both partners give written informed consent
5. Those having at least 3 2PN embryos (showing 2 pro-nuclei which is a sign of normal fertilisation) on day of fertilisation check

#### Exclusion criteria

1. Participants concomitantly participating in other interventional trials
2. IVF/ICSI treatment using donor gametes
3. Planned pre-implantation genetic diagnostics or screening (PGS/PGD)

The study is open to any sites willing to randomise participants to the 3 treatment groups, use standardised gas, temperature and media for all 3 incubation methods and for embryologists to adhere to specific quality control detailed below to maximise allocation adherence and maintaining the study blind.

### Who will take informed consent? {26a}

#### Screening

Potentially eligible participants at the recruiting IVF clinics will be identified at routine clinic appointments before the start of their IVF/ICSI treatment and will be given a letter of invitation to participate in the trial. Those interested will see a member of the research team who will give them detailed verbal information and a participant information sheet (PIS). Participants will have ample time to read the information leaflets, ask any questions and make an informed choice of whether to participate or not. A minimum of 24 h is recommended for couples to consider the trial, although well-informed participants can be consented sooner than that if the local research team believes they have a good understanding of the trial and their involvement in it. If participants agree to be a part of the trial, they will be consented at the next available opportunity.

#### Informed consent

Informed consent of both partners will be taken by a trained and delegated member of the study team. This will be done at their clinic appointment or subsequent visits to the clinic up until but prior to the egg collection procedure.

Consent will be confirmed by the doctor performing the egg collection on the day of the procedure, and this confirmation of consent will be documented in the woman’s medical notes. Some participants who have consented as they fulfil all other eligibility criteria will not be randomised to the trial due to an inadequate number of eggs or embryos, or because of insufficient capacity in all incubator types at the time of randomisation. Such participants may still participate in the trial in subsequent treatment attempts but will need to be consented again. In addition, those participants who were randomised previously can be consented and randomised again, as long as they continue to meet all trial inclusion criteria.

A copy of the consent form will be given to the participants; one copy will be kept in the woman’s hospital notes and one will be placed in the Investigator Site File. Only members of the research team documented on the delegation log will be able to consent eligible couples for participation in the study. The consenting staff will have thorough knowledge and documented training of research governance issues surrounding consent and will be fully conversant with the study protocol.

The qualified person taking consent must explain to the potential participants that they are free to refuse any involvement within the study or alternatively withdraw their consent at any point during the study and for any reason. If there is any further safety information that may result in significant changes in the risk/benefit analysis, the PIS and informed consent form (ICF) will be reviewed and updated accordingly. All participants that are actively enrolled in the study will be informed of the updated information and given a revised copy of the PIS and ICF in order to confirm their wish to continue in the study (if feasible), if it may change their willingness to participate. Participants who speak limited English can only be consented and included in the trial if translation has been provided by an independent translator (i.e. not a family member) or through the Language Line translation service for UK sites. International sites will translate patient-facing documentation into the required language.

### Additional consent provisions for collection and use of participant data and biological specimens {26b}

Not applicable as there are no ancillary studies planned for the trial.

## Interventions

### Explanation for the choice of comparators {6b}

The hypothesised benefit of the TLI systems may be due to either a closed undisturbed culture system or the use of morphokinetic parameters for embryo selection or both. Hence, a three-arm study design is necessary to answer the research question and define the contributions of both the variables involved. The comparator for both the intervention groups 1 and 2 (TLI and undisturbed culture) will be standard care, i.e. embryo incubation using a conventional, non-TLI incubator and embryo selection based solely on morphological assessment, and necessitating removal of the embryo from the incubator for examination under a light microscope. This is the ideal comparator as allows comparison to groups 1 and 2 in terms of (a) efficacy of morphokinetic parameters given by time-lapse imaging and (b) efficacy of undisturbed culture provided by the time-lapse incubator. To date, this method is seen as the standard of care in the absence of evidence that time-lapse imaging provides superior results.

### Intervention description {11a}

#### Care pathway

Participants will start their IVF/ICSI treatment and will follow the steps for the standard care pathway to the point of IVF or ICSI. This will include controlled ovarian stimulation with exogenous hormones, final maturation trigger for the release of eggs, sperm production, egg collection with a minor surgical procedure and the procedure of IVF or ICSI as indicated. Media and all other standard laboratory conditions will vary with recruiting centres but will remain the same for all three arms within the particular centre. Consent for the trial will be taken at clinic appointments or subsequent visits to the clinic up until but prior to the egg collection procedure.

Differences in the care pathway/trial intervention will involve the steps of embryo incubation, assessment and selection for transfer into the womb. Following the trial intervention, all further care, which includes embryo transfer, pregnancy test and review appointments, will be according to the standard care pathway.

#### Eligibility check

Prior to randomisation, a final eligibility check will be carried out. Participants having IVF or ICSI will need at least 3 2PN embryos (showing 2 pro-nuclei which is a sign of normal fertilisation) on the day of fertilisation check. Randomisation will be done only subject to availability of a space in incubators for each arm of the study.

#### Ineligible and non-randomised participants

Some participants who have been consented will not be eligible for randomisation due to inadequate numbers of eggs or embryos. A member of the research team will inform these participants that they have not fulfilled the eligibility criteria. These participants will continue to have standard care according to local protocols.

#### Trial intervention/allocation

Participants who have at least three fertilised embryos (2 pro-nuclei) on the day after egg collection will be randomly allocated to one of the following three groups:

1. Participants in the first/time-lapse imaging arm (intervention 1) will have embryo assessment and scoring with the morphokinetic parameters obtained from the time-lapse imaging system in addition to the standard morphological embryo scoring systems in undisturbed culture conditions in the time-lapse imaging incubators.
2. Those in the second/undisturbed culture arm (intervention 2) will have embryo assessment obtained using only conventional morphological embryo assessment in undisturbed culture conditions in the time-lapse imaging incubators. No morphokinetic assessment will be performed in this group.
3. Those in the third/standard care arm (control) will have an embryo assessment obtained using only conventional morphological embryo assessment using the light microscope and standard embryo culture in standard incubators.

Each recruiting centre may use a TLI system of their choice. However, the same TLI system will be used in both intervention arms 1 and 2 at each individual centre, either as a high-quality incubator providing undisturbed culture condition alone or with the addition of the morphokinetic parameters.

#### Laboratory procedures

##### Egg collection and fertilisation

Egg collections will be performed between 35 and 38 h after the ovulation trigger injection. These eggs will be kept in the standard incubators until the next procedure which is insemination for IVF or removal of cumulus cells and injection of sperm for ICSI. Timing of the ICSI or the standard insemination will vary depending on the time of fertilisation check of the laboratory. Fertilisation checks will be completed, and the fertilised oocytes/embryos will be placed in the appropriate incubators based on randomisation between 16 and 19 h from the ICSI/insemination procedure. As all embryos will be in standard incubators at the time of fertilisation check, the is important and specified to make sure that the annotations for the time-lapse incubator start from PN fading to get maximum information for embryo selection.

##### Standardised settings for incubators

Although the laboratories can follow their own centre protocols, they should make sure that there are no other variables between the study groups other than the study intervention. Particular attention should be given to the following:

- The culture media used in all three trial groups to be the same.
- Time-lapse incubators use triple gas and therefore the standard incubators used in the trial have to be on triple gas as well. In addition, the gas concentrations are to be the same (CO_2_, O_2_ and N_2_) in all the incubators. The actual gas concentrations may vary from centre to centre depending on the culture medium used and the local protocols. However, the concentrations need to be the same across all three arms and in all trial incubators.
- All incubators used in the trial to be set at the same temperature.
- All the oocytes from participants consented for the trial have to be kept in the standard incubator on the day of egg collection after ICSI or insemination. (If any patient has only one or two oocytes injected or inseminated, then they are not eligible for the trial, and therefore, the lab can culture them in any incubator).
- On day 1 after egg collection, the fertilised eggs are allocated to the appropriate incubators according to the randomisation. (If any patient has only one or two oocytes fertilised, then they are not eligible for the trial, and the lab can culture them in any incubator.)

##### Documentation of allocation

Once randomisation is performed, this information should not be stated in the patient notes or any document that goes into the patient notes as all staff except embryologists are blinded for the trial. Embryologists should have a separate randomisation sheet that can be kept in the lab only.

##### Embryo grading

The embryo grading will be performed on day 3 and day 5 (and, if needed, on day 6). The morphological grading on day 3 will take account of the number of cells/blastomeres, the regularity of blastomeres and the degree of fragmentation. The grading on day 5 will be based on the expansion of blastocyst, inner cell mass and trophectoderm. The Association of Clinical Embryologists (ACE) embryo grading scheme introduced in April 2017 will be followed to grade the embryos on day 3 and day 5.

##### Embryo selection

The selection of embryos for transfer will be as follows:

- For embryos in all three treatment groups, all the available embryos will be graded on the basis of morphology.
- For embryos in treatment group 1, individual labs will then apply and document morphokinetic parameters and any other information available from the time-lapse imaging, according to local policy, and select the embryos for transfer.
- Embryo selection will be cross-checked by a second embryologist on the day of transfer/freezing to ensure allocation selection is done in adherence with the randomised allocation. This cross-check will be documented in the study database.

##### Embryo transfer

Randomised patients for fresh embryo transfer will have their embryos transferred into the uterus on either day 3 or day 5 after the egg collection (or day 6 if applicable). The participating centre can decide the day of embryo transfer and the number of embryos to be transferred according to their local protocols. Randomised patients for frozen embryo transfer will have their embryos frozen either on day 3 or day 5 after the egg collection. The participating centre can decide the day of freezing according to their local protocols. The selection of embryos for subsequent transfer is done prior to freezing, as per the randomised allocation. The frozen embryos selected for subsequent transfer are to be clearly marked to ensure correct identification.

### Criteria for discontinuing or modifying allocated interventions {11b}

Participants will be able to withdraw their consent to take part in the trial at any time without giving a reason. Given the short duration and low risk of the intervention, it is not foreseen that participants would need to be withdrawn or have their treatment modified during their study participation. However, clinicians may withdraw participants from the trial if they feel it is in the participant’s best interest. Withdrawal from the trial will not affect their ongoing care. If consent is withdrawn, data already collected up to the point of withdrawal will be retained (in line with the UK Data Protection Act 2018) and permission will be sought to complete follow-up outcomes data collection. If participants withdraw their consent while embryos are still incubating (days 2–6), couples will be advised to allow their embryos to remain in the allocated incubator to minimise any disturbance to the embryos.

### Strategies to improve adherence to interventions {11c}

Participant adherence is not applicable in this trial. The only risk of non-adherence is linked to the use of identical devices for both group 1 (TLI) and group 2 (undisturbed culture), meaning time-lapse imaging would be available for inspection in group 2. This will be mitigated as follows:

- Prior to being involved in the trial, embryologists will receive study-specific training and will sign off training documentation confirming their agreement to adherence to allocation and retaining blinding.
- Assessment of morphokinetic parameters during incubation of group 1 (TLI) embryos is always documented in lab notes and can generally be tracked through ongoing annotations of time-lapse imaging, and this provides an audit trail that is available for monitoring compliance.
- Before embryo transfer or freezing, a second embryologist will cross-check and confirm the selection based on the allocated treatment—i.e. based on TLI for group 1 participants, and based on static image assessment only for group 2 participants.
- Any non-adherence will be elicited and documented on study CRFs.

### Relevant concomitant care permitted or prohibited during the trial {11d}

The design of this trial will be pragmatic. It will allow centres to use a TLI system of their choice. The trial tests the basic premise that undisturbed culture and/or addition of morphokinetic parameters to standard embryo assessment improves outcomes in IVF/ICSI treatment. It does not aim to test and validate individual TLI systems and algorithms. It is accepted that clinical protocols for IVF and laboratory conditions will vary with each centre. All clinical and laboratory elements of IVF/ICSI treatment apart from the randomised intervention will be carried out according to local centre protocols, ensuring only that there is no variation across the three study arms in temperature, culture medium and use of gas. There is no contraindication to any other standard treatment; however, to ensure robust methodology, the trial will exclude participants who are concomitantly participating in other interventional trials, or have planned pre-implantation genetic diagnostics or screening (PGS/PGD), as this would interfere with the embryo selection process.

### Provisions for post-trial care {30}

Following the trial intervention, all further care, which includes embryo transfer, pregnancy test and review appointments, will be according to the standard care pathway.

The Queen Mary University of London is the sponsor for the UK arm of this trial. The university will obtain and hold insurance policies for legal liabilities arising from the trial. The recruiting sites are NHS units and have indemnity arrangements in place which will cover their liabilities in relation to their participation in the study.

The sponsor of international sites will cover their own indemnity for this trial.

### Outcomes {12}

#### Primary outcome

The primary outcome is live birth.

#### Secondary outcomes

Clinical efficacy outcomes:

1. Pregnancy rate (positive pregnancy test approximately 2 weeks after embryo transfer) per participant randomised
2. Successful clinical pregnancy rate (at least one intrauterine gestational sac seen at 6–8 weeks of gestation; multiple pregnancy counts as one clinical pregnancy) per participant randomised
3. Clinical pregnancy rate per embryo transferred (total number of gestational sacs seen on ultrasound scan/total number of embryos replaced into the womb)
4. Use of elective single embryo transfer (e-SET) per participant randomised
5. Embryo utilisation rate (% of total embryos either transferred or frozen)

#### Clinical safety outcomes

1. Multiple pregnancy (two or more gestational sacs seen on ultrasound scan at 6–8 weeks) per clinical pregnancy
2. Pregnancy loss
  (i) Between positive pregnancy test and 6–8-week scan per positive pregnancy test
  (ii) Between 6- and 8-week scan and 12 weeks (early miscarriage) per clinical pregnancy
  (iii) Between 12 and 24 weeks per clinical pregnancy
  (iv) Stillbirth per clinical pregnancy
3. Incidence of major congenital abnormalities per participant randomised
4. Birth weight
5. Gestational age
6. Ectopic pregnancy per participant randomised

Live birth was chosen as the primary outcome as this remains the most relevant outcome for participants, clinicians and all stakeholders. Our secondary clinical efficacy and safety outcomes were chosen as these have the biological plausibility to be relevant to the trial intervention and also are identified as important core fertility outcomes to be reported in fertility trials.

### Participant timeline {13}

The trial intervention will be an alternative method for incubation and assessment of embryos created during IVF/ICSI. There will be no extra added intervention for the embryos. There will be no extra visits/intervention for the participants (Table 1).

**Table 1:** Schedule of assessments

	Baseline	Day of egg collection (D0)	Day of fertilisation check (D1)	Days 3, 5 and optionally day 6	Routine visit to fertility unit approx. 2 weeks after embryo transfer	Approx. 8 weeks of gestation	Approx. 24 weeks of gestation	Approx. 6 weeks after the expected due date
Informed consent	X							
Confirmation of informed consent		X						
Baseline demographic and clinical data	X							
Randomisation and confirmation to randomised participants			X					
Ineligible consented participants informed			X					
Embryo grading; transfer if appropriate				X				
Documentation of morphokinetic parameters (arm 1 only)				X				
Pregnancy test outcome					X			
Elective single embryo transfer rate					X			
Clinical pregnancy rate						X		
Multiple pregnancy rate						X		
Miscarriage rate							X	
Live birth rate								X
Stillbirth rate								X
Multiple birth rate								X
Serious adverse events			X		X	X	X	X

### Sample size {14}

#### Sample size

The sample size calculation was based upon the primary outcome of live birth. With a 5% overall significance level (2.5% for each of the two main treatment comparisons: TLI vs. standard care, and undisturbed culture vs. standard care), we would require 514 participant randomisations per treatment arm to detect an absolute increase in the primary outcome from 26.5 to 35.25% with 80% power. Allowing for 2% loss to follow-up or withdrawal of consent would require 525 participants per treatment arm (1575 in total).

The comparison between experimental treatment arms (TLI vs. undisturbed culture) will be performed with no impact on sample size because we will carry out this statistical test conditional to the rejection of at least one of the primary comparisons planned (TLI vs. standard care, or undisturbed culture vs. standard care). This hierarchical approach permits to maintain the overall type I error rate of 5%.

This trial uses the re-randomisation design, which was described by Kahan et al. [9]. Requirements for the re-randomisation design are as follows:

1. Patients are only re-randomised when they have completed the follow-up period from their previous randomisation.
2. Randomisations for the same patient are performed independently.
3. The treatment effect is constant across all randomisation periods.

Requirement 1 is fully satisfied, as participants will have completed their previous round of treatment before re-joining the study. Participants have an equal chance of being randomised to either of the three trial arms, regardless of their previous randomisation result, therefore satisfying requirement 2. Finally, there is no anticipated treatment effect, either in terms of efficacy or safety, from one episode to the next.

It has been demonstrated that introducing re-randomisation in a trial will not affect the validity of the statistical analysis and results [9].

### Recruitment {15}

The trial poster will be displayed in fertility clinics and on the fertility pages of local trust websites for the purpose of advertising the trial. Trial information will also be given out at information evenings. Where suitable, Patient Identification Centres (PIC) will be utilised to identify patients referred to participating fertility clinics from secondary care fertility clinics.

## Assignment of interventions: allocation

### Sequence generation {16a}

Sequence generation for randomisation will be done using a secure web-based randomisation system immediately prior to randomisation and subsequent allocation of intervention.

The randomisation will be stratified by a fertility clinic and minimised by the following:

1. Participant’s age (< 35 years, 35–40 years, > 40 years)
2. Type of planned first embryo transfer (fresh, frozen).

Minimisation for both factors will include a 90% weighting to introduce 0.9 as a stochastic factor for allocation probability.

### Concealment mechanism {16b}

The allocation sequence will be implemented using a secure web-based randomisation system. The system will be tested prior to deployment using dummy sequences.

Access to the live randomisation system is given only to embryologists, who are the only staff unblinded to the intervention. Each embryologist at centres uses a personal, unique login and password to access the randomisation system. User access and allocation of the correct level of access is managed by the central trial team. Introduction of weighting of minimisation factors (as described above) will introduce a stochastic element that ensures allocation sequences remain unpredictable.

### Implementation {16c}

A secure web-based randomisation system will generate the allocation sequence. Randomisation will be performed on the day of fertilisation check. Participants satisfying all the eligibility criteria for the trial will be randomised into one of the three treatment arms in a 1:1:1 ratio.

1. Time-lapse imaging
2. Undisturbed culture
3. Standard treatment

Randomisation will be done by a trained and delegated embryologist member of the study team using a secure web-based randomisation system hosted by epiGenesys, University of Sheffield, which is accessible around the clock, 365 days of the year. Back-up procedures in case of technical issues with accessing the randomisation system will be made available and described in a study-specific standard operating procedure (SOP).

## Assignment of interventions: blinding

### Who will be blinded {17a}

It will not be possible to blind embryologists to the intervention. The trial statistician is ‘semi-blinded’ to the allocation—they will see data by trial arms for DMC report generation, but trial arms will not be identified. All other local and central trial staff, including clinicians performing the embryo transfer and data analysts, will be blinded. Participants will be blinded to the allocated intervention until the end of their participation in the trial. This will either be a negative pregnancy test which is approximately 2 weeks after embryo transfer or the end of their pregnancy. Once randomisation is performed, embryologists will document the allocation in a local randomisation form that will remain in the lab to prevent unblinding and proceed with the intervention and laboratory procedures described below. A randomisation log will be kept in the lab and accessed and updated by study embryologists only.

### Procedure for unblinding if needed {17b}

Local delegated embryologists will be the holders of the code break list for each site. Given the low risk of the intervention, it is unlikely that urgent unblinding should be required to guide patient management. In the unlikely event that an emergency codebreak is requested, the PI or healthcare professional will request unblinding from the embryologist. On receipt of the treatment allocation details, the PI or treating healthcare professional will continue to deal with the participant’s medical emergency as appropriate. The PI must document the breaking of the code and the reasons for doing so on the CRF/data collection tool, in the site file and medical notes.

Additionally, participants may request to be informed of their allocation when they reach the end of their study participation (either due to unsuccessful pregnancy or end of pregnancy). Care will be taken not to disclose the allocation to any other member of the research team.

Any unblinding, including accidental unblinding (which constitutes a protocol deviation), will be reported to the Data Monitoring Committee (DMC) and included in the final study report.

## Data collection and management

### Plans for assessment and collection of outcomes {18a}

Trial data will be recorded by the research team at each of the participating centres directly onto trial-specific secure password-protected electronic case report forms (eCRFs) in a database administered by epiGenesys, University of Sheffield. Data that is entered directly by the embryologists (randomisation data and intervention data) will be restricted to allow access only to unblinded staff members.

The following data will be collected:

1. Baseline demographic data (age, ethnicity)
2. Baseline clinical data (category of infertility, type of infertility, duration of infertility, BMI, treatment attempt, parameters of egg reserve and sperm count)
3. Clinical treatment data (stimulation protocol, drug dosages, duration of stimulation, number of oocytes retrieved, type of insemination procedure, number of embryos available, fresh/frozen transfer, day of embryo transfer, number of embryos transferred and type of time-lapse machine, embryo grading; if applicable, documentation of morphokinetic parameters will be documented and submitted separately by embryologists to maintain blinding of the remaining study team)
4. Data for all clinical outcomes (live birth, pregnancy test outcome, implantation, clinical pregnancy, multiple pregnancy, miscarriage, stillbirth, neonatal data)

For all participants, clinical and laboratory data are routinely recorded in the fertility medical notes or fertility electronic database by clinical staff. If clinical outcome data are not available in the medical records, a trained delegated member of the research team will phone the participants to obtain this data. Trained and delegated members of the trial team, as documented on the trial delegation log, will be responsible for the completion of the eCRFs.

### Plans to promote participant retention and complete follow-up {18b}

If consent is withdrawn, data already collected up to the point of withdrawal will be retained, and permission will be sought to complete follow-up outcomes data collection. The duration of the intervention is short, and follow-up procedures are part of standard care due to HFEA reporting requirements; hence, we do not anticipate issues with follow-up.

### Data management {19}

All data management will be undertaken by the Queen Mary University of London. Standard operating procedures will be in place for the collection and handling of data received at the centre. All study data will be entered into a secure, bespoke electronic trial database with restricted access. Data collected on the data collection forms and entered onto the electronic database will only identify the participants by a unique trial number.

Data will be processed on a workstation by authorised staff. The workstations access the network via a login name and password. No data are stored on individual workstations. Backing up is done automatically overnight to an off-site storage area. Sites will allow access to source data and documentation for the purpose of monitoring, auditing and inspections, for the relevant authorised individuals.

### Confidentiality {27}

The investigator has a responsibility to ensure that patient anonymity is protected and maintained. They must also ensure that their identities are protected from any unauthorised parties. Information with regard to the study patients from UK sites will be kept confidential and managed in accordance with the Data Protection Act (2018), NHS Caldecott Guardian, principles, The Research Governance Framework for Health and Social Care, and Research Ethics Committee Approval. International sites will abide by their own countries regulatory requirements.

The trial will collect personal data and sensitive information about the participants either directly or from their clinical team. Participants will be informed about the transfer of this information to the study office and will be asked to consent to this. The data will be entered onto a secure computer database, either by trials unit staff or directly via a secure internet connection. Any data to be processed will be pseudo-anonymised. At recruitment, eCRFs will be pseudonymised using a unique participant code that is allocated by the recruiting member of staff. At randomisation, an additional unique code will be generated by the online randomisation system and recorded on the randomisation form. All personal information obtained for the trial will be held securely and treated as strictly confidential. All staff, at each hospital and the trials unit, share the same duty of care to prevent unauthorised disclosure of personal information. No data that could be used to identify an individual will be published. The chief investigator, Dr. Priya Bhide, is the ‘custodian’ of the data.

### Plans for collection, laboratory evaluation and storage of biological specimens for genetic or molecular analysis in this trial/future use {33}

Not applicable as no biological specimens will be collected as a part of this trial.

## Statistical methods

### Statistical methods for primary and secondary outcomes {20a}

Analyses will be intention-to-treat, will include all randomised participants and will analyse according to the treatment group to which they were randomised. We will analyse each participant randomisation as an independent observation (i.e. perform an independence analysis). As a sensitivity analysis, we will provide results from analyses restricted to complete cases, for comparison with results based on multiple imputation.

For each analysis, we will present the treatment effect (difference in means for a continuous outcome and an odds ratio for binary outcomes) along with a 95% confidence interval and a two-sided *p* value. Because we have two main treatment comparisons (TLI vs. standard care, and undisturbed culture vs. standard care), the significance level for the primary outcome is set at 2.5% for each treatment comparison. The primary outcome (live birth) will be analysed using logistic regression model and will be adjusted for stratification and minimisation factors and other pre-specified covariates to increase power. The stratification factor is the fertility clinic and the minimisation factors are participant’s age (< 35 years, 35–40 years, > 40 years) and type of planned first embryo transfer (fresh, frozen). Other covariates to be included in the analyses are treatment attempt, type of infertility (primary/secondary), category of infertility, duration of infertility, BMI, type of time-lapse imaging equipment, method of insemination (IVF vs. ICSI), number of retrieved oocytes and number of available embryos. A full statistical analysis plan will be developed and finalised prior to data analysis and will include full specifications on subgroup analyses.

### Interim analyses {21b}

No interim analyses or early stopping rules are foreseen.

### Methods for additional analyses (e.g. subgroup analyses) {20b}

A full statistical analysis plan will be developed and finalised prior to data analysis and will include full specifications on subgroup analyses.

### Methods in analysis to handle protocol non-adherence and any statistical methods to handle missing data {20c}

Analyses will be intention-to-treat, will include all randomised participants and will analyse according to the treatment group to which they were randomised.

Missing data (for independent variables and outcome data) will be imputed using multiple imputation via chained equations.

### Plans to give access to the full protocol, participant level-data and statistical code {31c}

The trial summary is available in the public domain on the ISRCTN registry.

Data sharing: Following primary publication of the trial results, fully anonymised trial data and statistical analysis code can be made available to interested researchers, where appropriate, on request to the Chief Investigator.

## Oversight and monitoring

### Composition of the coordinating centre and trial steering committee {5d}

#### Trial management

The UK arm of the study is sponsored by the Queen Mary University of London (QMUL) and conducted by the Barts Research Centre for Women’s Health (BARC). International sites will have their own separate sponsor. QMUL will also have main oversight on a global level. The trial will be coordinated and managed on a day-to-day basis by the Trial Management Group (TMG) comprising of core members from the co-applicants and the BARC. The Co-Investigators Group (CIG), Data Monitoring Committee (DMC) and Trial Steering Committee (TSC) will provide strategic direction. Appointment of the TSC and DMC will remain the responsibility of the TMG.

#### Trial Management Group (TMG)

The TMG will comprise the chief investigator, co-investigators, senior trials manager, trial coordinator, embryology representative, clinical trial practitioner and statistician. The TMG will be based at QMUL and meet monthly. They will have overall responsibility for the conduct of the trial and will report to the TSC.

#### Trial Steering Committee

The trial will be supervised by a single international independent Trial Steering Committee (TSC). The TSC will have an independent chair and at least two further independent members, such as a clinician, embryologist and a PPI representative. The TSC will meet biannually. The CI, co-applicants, trials coordinator and senior trials manager will be invited to attend the TSC meetings. The specific tasks of the TSC will be:

- To recommend and approve major amendments to the protocol arising during the trial
- To receive the reports from the TMG and DMC
- To approve the statistical analysis plan and any changes thereto
- To resolve problems brought to it by the trial collaborators
- To review trial reports and the main paper for publication

#### Co-Investigators Group

The Co-Investigators Group (CIG) will comprise the chief investigator, co-investigators, key staff from QMUL and principal investigators and key staff from the recruiting centres. They will provide strategic direction to the trial and will meet twice during the course of the trial, during recruitment and in the data analysis phase.

The clinical trial report will be submitted to the CIG and TSC for review before publication.

The timing, content and remit of the committee meetings will be decided at the first meeting.

### Composition of the data monitoring committee, its role and reporting structure {21a}

A single international independent Data Monitoring Committee (DMC) will be established for the trial. It will comprise of an independent chair and at least two further independent members who are experts in the field, such as a clinician, trial methodologist and statistician. The DMC will meet biannually. Collaborators and all others associated with the trial may write through the trial office to the DMC, to draw attention to any concern they may have about the possibility of harm arising from the treatment under study, or about any other matters that may be relevant. The DMC has the right to review unblinded data reports. The DMC will also monitor the progress of the trial and will report to the TSC.

### Adverse event reporting and harms {22}

#### Adverse events (AE)

An AE is any untoward medical occurrence in a trial participant, including occurrences which are not necessarily caused by or related to the trial intervention. An AE can therefore be any unfavourable and unintended sign (including an abnormal laboratory finding), symptom or disease temporarily associated with trial activities.

Due to the high incidence of adverse events routinely expected in this patient population and the low risk of the intervention, only those adverse events identified as serious will be recorded for the trial.

#### Serious adverse event (SAE)

A serious adverse event (SAE) is defined as an untoward occurrence that:

- Results in death
- Is life-threatening
- Requires hospitalisation or prolongation of existing hospitalisation, apart from the following events which are foreseeable in pregnant women undergoing IVF/ICSI and hence will not require reporting as SAEs:
  Events relating to the participant:
  1. Mild/moderate ovarian hyperstimulation syndrome (not requiring paracentesis)
  2. Pelvic infection/pelvic inflammatory disease
  3. Multiple pregnancy
  4. Miscarriage
  5. Ectopic pregnancy
  6. Any other hospitalisations for known pregnancy complications
  7. Any other hospitalisations for known postpartum complications
  8. Any hospitalisations for labour
  Events relating to the baby:
  1. Low/very low birth weight
  2. Small/large for gestational age
  3. Preterm/very preterm delivery
- Results in persistent or significant disability or incapacity
- Consists of a congenital anomaly or birth defect
- Is otherwise considered medically significant by the investigator.

N.B. Any event requiring admission to the neonatal intensive care unit (NICU) other than those listed above is to be reported as SAEs.

All congenital anomalies are to be reported as SAEs, whether identified at birth or earlier (e.g. cause for miscarriage or termination).

Events that fulfil the criteria for seriousness and are not amongst the above list will need to be reported as SAEs. They also need to be assessed as to whether they are (possibly, probably or definitely) related to administration of trial procedures.

Any SAEs that are (possibly, probably or definitely) related to the trial procedures will also need to be assessed as to their expectedness. As there are no expected adverse effects of TLI, any events that are considered by the local investigator to be related to the use of a TLI device will be classed as unexpected.

#### Notification and reporting of serious adverse events

Data on SAEs will be documented from randomisation, at the scheduled outcome assessment points from the medical records, and up to 6 weeks post-delivery in case of successful pregnancies. This may include telephone contact with the mother approximately 6 weeks after delivery if required. Research staff should refer to all available resources including medical records, online systems such as CRS, and discussions with participants themselves to ensure that all reportable SAEs are elicited.

Any SAEs that require reporting need to be reported by the local PI to the chief investigator (CI) within 24 h of learning of the event. The CI will assess the event and can always upgrade the relationship or severity of the event, but never downgrade it. The CI will assess if a ‘related’ SAE was ‘unexpected’. Serious adverse events (SAEs) that are considered to be ‘related’ and ‘unexpected’ are then to be reported to the sponsor within 24 h of learning of the event and to the Research Ethics Committee (REC) within 15 days in line with the required timeframe. International sites will report to the QMUL sponsor in addition to their own sponsor.

#### Other safety considerations and reporting

The CI may need to take urgent safety measures to ensure the safety and protection of the clinical trial subjects from any immediate hazard to their health and safety. The measures will be taken immediately. In this instance, the approval of the REC prior to implementing these safety measures is not required.

The CI will send the annual progress report to the main REC on the anniversary date which is the date of the REC favourable opinion, as well as to the sponsor.

The DMC will review reports of ‘related’ and ‘unexpected’ SAE. If appropriate, it will make recommendations for the continuance of the trial or modification of the study protocol.

### Frequency and plans for auditing trial conduct {23}

The sponsor has assessed the study as low risk for the purpose of quality control. The study sites will therefore perform trial self-monitoring according to the agreed trial monitoring plan and self-monitoring template. Trial monitoring will include source data verification checks on informed consent forms and eligibility for randomisation and a sample set of CRFs. The self-monitoring reports will be reviewed by QMUL and all findings will be followed up and actioned as per the trial monitoring plan. The study sites will return self-monitoring templates to QMUL every 6 months.

In addition, central data monitoring will be performed by the central trial team, by identifying outliers and missing data through regular data monitoring reports. Central data administration staff will issue data queries to sites.

The sponsor has the right to carry out an internal audit throughout the duration of the trial.

A study may be identified for audit by any method listed below:

1. A project may be identified via the risk assessment process.
2. An individual investigator or department may request an audit.
3. A project may be identified via an allegation of research misconduct or fraud or a suspected breach of regulations.
4. Projects may be selected at random. The Department of Health states that Trusts should be auditing a minimum of 10% of all research projects.
5. Projects may be randomly selected for audit by an external organisation.

Audits of UK sites will be conducted by a sponsor’s representative.

### Plans for communicating important protocol amendments to relevant parties (e.g. trial participants, ethical committees) {25}

The trial can only start after approval from a REC, the UK Health Research Authority (HRA) and confirmation of local capacity and capability at each of the participating centres (or equivalent ethics committee bodies for international sites). If there is any further safety information which may result in significant changes in the risk/benefit analysis, the protocol, PIS and ICF will be amended accordingly and submitted to the REC for revision and approval. All participants that are actively enrolled on the study will be informed of the updated information and given a revised copy of the PIS/ICF in order to confirm their wish to continue on the study (if feasible), if it may change their willingness to participate.

### Dissemination plans {31a}

1. The CI will have primary responsibility and coordinate dissemination of data from this trial. A core team consisting of the co-investigators will work closely with QMUL to plan and effectively disseminate the findings of the research to all stakeholders: participants, clinical community, user groups, funding bodies, NHS commissioners and the general public. The clinical trial report and the main manuscript will be reviewed by the CIG and TSC before publication.
2. Dissemination to clinicians and clinical professional bodies will be through publications and presentations at major national and international conferences relevant to the speciality. We aim to publish the findings in the highest impact peer-reviewed journals and present them at the annual conferences related to the speciality. We plan to publish the study protocol in an open-access journal and to communicate the trial findings to the Cochrane Gynaecology and Fertility Group with a view to incorporate the results into the current Cochrane review.
3. Dissemination to the participants and the general public will be done through newsletters, NHS websites and through the meetings and websites of local PPI networks and Fertility Networks UK. In consultation with the investigators and appropriate journals, a press release will be issued to the media upon publication of the results.
4. A writing committee will be appointed which will follow the authorship criteria used by high-impact peer-reviewed journals (www.icjme.org). Members of the committee will be named authors on the trial monograph and principal study paper. Other team members with substantial contribution to the trial will be formally acknowledged in publications arising from the trial.
5. There is currently no national guidance on the use of time-lapse imaging in IVF/ICSI. The NICE guideline development process will be informed of the results of the trial, which will be important for their guideline updates.
6. Time-lapse imaging is currently not incorporated into national IVF/ICSI tariffs. The costing of IVF may need to be revised in order to include the costs of time-lapse imaging into routine NHS funded IVF/ICSI care. We plan a dissemination event involving the NHS commissioners and funding stakeholders with a view to incorporating the findings of the study into NHS funding, practice and policy.
7. Data sharing: following the primary publication of the trial results, fully anonymised trial data can be made available to interested researchers on request to the chief investigator and approval from the sponsor.

## Discussion

Time-lapse imaging of embryos during IVF/ICSI treatment is a newer technology hypothesised to improve outcomes of treatment. This may be due to the provision of undisturbed culture conditions for the embryos and/or improving the predictive accuracy of embryo selection. The presence of two variables necessitates a three-arm trial. However, the current best evidence for the use of time-lapse imaging in IVF/ICSI is inconclusive. Previous RCTs have typically used the clinical pregnancy rate as a surrogate outcome for the live birth rate, which remains the most relevant outcome for patients, clinicians, and policymakers. Furthermore, the long-term safety of this procedure is unknown. The above-mentioned Cochrane reviews as well as the consensus from our preparatory surveys amongst clinicians, embryologists and patients have shown that a definitive conclusive trial is necessary.

TLI in IVF/ICSI is commercially available, extensively marketed and widely introduced in IVF clinics in spite of inconclusive evidence regarding its effectiveness and its considerable additional expense to clinics. It is a worrying trend to see a newer but expensive technology being introduced very rapidly into practice without high levels evidence of its clinical and cost-effectiveness or safety. In the absence of high-quality evidence and slipping clinical equipoise, it is necessary to urgently perform a large randomised controlled trial to confirm or refute the benefit of time-lapse imaging in IVF/ICSI.

This trial will also generate a dataset which will remain a useful resource for future health economic assessment and assessment of maternal and perinatal outcomes. Furthermore, the trial will create a cohort of children exposed to the intervention from which long-term safety data may be collated through follow-up studies conducted in the future.

## Trial status

The current version of the study protocol is v7.0, 04 September 2019. Recruitment began on 27 June 2018 and the recruitment target is expected to be reached by 28 February 2021.

## Supplementary information

**Additional file 1.** CONSORT diagram.

## Abbreviations

ACE: Association of Clinical Embryologists
AE: Adverse event
BARC: Barts Research Centre for Women’s Health
BMI: Body mass index
CI: Chief investigator
CIG: Co-Investigators Group
CRF: Case report form
DET: Double embryo transfer
DMC: Data Monitoring Committee
e-SET: Elective single embryo transfer
GCP: Good Clinical Practice
HFEA: Human Fertilisation and Embryology Authority
HRA: Health Research Authority
ICF: Informed consent form
ICSI: Intracytoplasmic sperm injection
IVF: In vitro fertilisation
JRMO: Joint Research Management Office
KID: Known implantation data
MRC: Medical Research Council
NEQAS: National External Quality Assessment Service
NHS REC: National Health Service Research Ethics Committee
NHS R&D: National Health Service Research & Development
NICE: National Institute for Health and Care Excellence
OR: Odds ratio
PI: Principal investigator
PIS: Participant information sheet
PN: Pro-nuclei
QA: Quality assurance
QMUL: Queen Mary University of London
RCT: Randomised controlled trial
REC: Research Ethics Committee
SAE: Serious adverse event
SOP: Standard operating procedure
TLI: Time-lapse imaging
TMG: Trial Management Group
TSC: Trial Steering Committee
