# Supplementary material for: TILT: Time-Lapse Imaging Trial—a pragmatic, multi-centre, three-arm randomised controlled trial to assess the clinical effectiveness and safety of time-lapse imaging in in vitro fertilisation treatment
Source: Trials. 2020 Jul 1;21:600. doi: 10.1186/s13063-020-04537-2 (PMC7329433; doi:10.1186/s13063-020-04537-2)
Supplement: Supplementary file 1 — Additional file 1. CONSORT diagram. [file 13063_2020_4537_MOESM1_ESM.docx]

Delivery (n = )

- No embryos available for transfer ()

- Negative pregnancy test ()
- Pregnancy loss before scan ()

Excluded (n = )

- Treatment abandoned during stimulation ()
- Consent withdrawn ()
- Other ()

End of pregnancy (n = )

- No gestational sac seen on scan ()
- Miscarriage ()
- Termination ()

End of pregnancy (n = )

- Stillbirth ()

24-week assessment (n = )

6-8 week scan (n = )

2-week pregnancy test (n = )

Treatment Arm 1 (n = ….)

Treatment Arm 3 (n = ….)

Treatment Arm 2 (n = ….)

Excluded (n = )

- < 3 2PN embryos for transfer
- Insufficient eggs collected
- Lack of incubator space

Randomised (n = )

Participants recruited (n = )

Assess for eligibility after egg collection
